# Upregulation of PPARβ/δ Is Associated with Structural and Functional Changes in the Type I Diabetes Rat Diaphragm

**DOI:** 10.1371/journal.pone.0011494

**Published:** 2010-07-08

**Authors:** Nadège Salvi, Aziz Guellich, Pierre Michelet, Alexandre Demoule, Morgan Le Guen, Laure Renou, Gisèle Bonne, Bruno Riou, Olivier Langeron, Catherine Coirault

**Affiliations:** 1 UMRS INSERM 956, Institut de Myologie, IFR14, Université Pierre et Marie Curie-Paris 6, Paris, France; 2 UMRS INSERM 974, Institut de Myologie, IFR14, Université Pierre et Marie Curie-Paris 6, Paris, France; 3 UMR CNRS 7215, Institut de Myologie, IFR14, Université Pierre et Marie Curie-Paris 6, Paris, France; 4 Department of Emergency Medicine and Surgery, Groupe hospitalier Pitié-Salpêtrière, Assistance Publique-Hôpitaux de Paris (APHP), Paris, France; 5 Department of Anesthesiology and Critical Care, Groupe hospitalier Pitié-Salpêtrière, Assistance Publique-Hôpitaux de Paris (APHP), Paris, France; 6 Department of Pneumology, Groupe hospitalier Pitié-Salpêtrière, Assistance Publique-Hôpitaux de Paris (APHP), Paris, France; 7 Department of Metabolic Biochemistry, Groupe hospitalier Pitié-Salpêtrière, Assistance Publique-Hôpitaux de Paris (APHP), Paris, France; 8 Department of Physiology, Hôpital de Bicêtre, APHP, Le Kremlin-Bicêtre, France; University of Pittsburgh, United States of America

## Abstract

**Background:**

Diabetes mellitus is associated with alterations in peripheral striated muscles and cardiomyopathy. We examined diaphragmatic function and fiber composition and identified the role of peroxisome proliferator-activated receptors (PPAR α and β/δ) as a factor involved in diaphragm muscle plasticity in response to type I diabetes.

**Methodology/Principal Findings:**

Streptozotocin-treated rats were studied after 8 weeks and compared with their controls. Diaphragmatic strips were stimulated *in vitro* and mechanical and energetic variables were measured, cross bridge kinetics assessed, and the effects of fatigue and hypoxia evaluated. Morphometry, myosin heavy chain isoforms, PPAR α and β/δ gene and protein expression were also assessed. Diabetes induced a decrease in maximum velocity of shortening (−14%, P<0.05) associated with a decrease in myosin ATPase activity (−49%, P<0.05), and an increase in force (+20%, P<0.05) associated with an increase in the number of cross bridges (+14%, P<0.05). These modifications were in agreement with a shift towards slow myosin heavy chain fibers and were associated with an upregulation of PPARβ/δ (+314% increase in gene and +190% increase in protein expression, P<0.05). In addition, greater resistances to fatigue and hypoxia were observed in diabetic rats.

**Conclusions/Significance:**

Type I diabetes induced complex mechanical and energetic changes in the rat diaphragm and was associated with an up-regulation of PPARβ/δ that could improve resistance to fatigue and hypoxia and favour the shift towards slow myosin heavy chain isoforms.

## Introduction

Diabetes mellitus may impair respiratory function by reducing vital capacity, peak trans-diaphragmatic pressures, maximum voluntary ventilation, and respiratory muscle endurance in humans [1], [2]. However the precise mechanisms of these changes remain a matter of debate since several mechanisms related to diabetes neuropathy and/or myopathy may be involved in respiratory function impairment [3]. Moreover, peripheral airway involvement cannot be excluded [3] which, in association with complex changes in neuroventilatory drive and perception of dyspnea [4], may preclude a precise analysis of respiratory muscle function *in vivo*. The duration of diabetes and the indirect role of its major complications may also markedly interfere with these changes and these important points have been rarely taken into account. A number of biochemical enzymatic and functional changes in skeletal muscles, including respiratory muscles, have been reported in diabetes mellitus including altered carbohydrate and lipid metabolism [5]–[7], oxidative stress [8], and changes in membrane electrophysiology [9]. Van Lunteren et al. [10] recently reported an increased expression in genes involved in lipid metabolism, oxidative stress, and protein ubiquitination associated with a decreased expression in gene involved in carbohydrate metabolism. However, few studies have directly assessed the intrinsic properties of diaphragmatic muscle in diabetes mellitus [11] and some of them are difficult to interpret since they followed only a very short exposure to diabetes [8].

We examined diaphragmatic functional modifications induced by diabetes in the rat, including mechanics, energetics, and cross-bridges kinetics, under baseline condition and during fatigue and hypoxia, using the well-known model of streptozotocin-induced type I diabetes. Diaphragm histological structure and myosin heavy chain (MHC) composition were also analyzed. Because members of the nuclear hormone receptor superfamily, namely peroxisome proliferator-activated receptor (PPAR) have critical role in lipid and glucose metabolism and homeostasis, skeletal muscle fiber type expression and endurance [12], and in diabetic myocardium remodelling [13], [14], we hypothesized that changes in PPAR expression contribute to functional and structural changes in diabetic diaphragm. We focussed on PPAR α and β/δ the main isotypes expressed in the rat diaphragm [15]. Diaphragm histological structure and myosin heavy chain (MHC) composition were also analysed.

In summary, our data suggest that up-regulation of PPARβ/δ is associated with functional and structural changes induced by type I diabetes in the diaphragm.

## Methods

Care of the animals conformed to the officials edict presented by the French Ministry of Agriculture (Paris, France) and the recommendations of the Helsinki Declaration. Thus, these experiments were conducted in authorized laboratories (UMRS INSERM 956, UMR CNRS 7215, UMRS INSERM 974) and under the supervision of authorized researchers (permit numbers 75–636 and 75–786). Rats were maintained on a 12∶12-h light dark photoperiod and received rat chow and water *ad libitum*.

### Animals

Six-week old male Wistar rats (body weight 212±5 g, Charles River Laboratories, France) were assigned either to control group (n = 12) and diabetic group (8 weeks after streptozotocin administration, n = 12). Diabetes was induced by intravenous injection of streptozotocin (65 mg/kg; Sigma Chemical, l'Isle Abeau Chesnes, France) in the femoral vein [16], [17]. Transcutaneous determination of glucose blood concentration (Glucotrend®; Boehringer, Manheim, Germany) was performed to ensure that the animals became diabetic (*i.e.*, glucose blood concentration >25 mM/l). At the moment of euthanasia, blood samples were withdrawn and centrifuged at 5,000 g for 15 min; then plasma fractions were collected and stored at −20°C for further determination of non-fasting glucose blood concentrations (Cobas Integra 400; Roche Diagnostic, Manheim, Germany).

### Mechanical and energetic parameters

After a brief anesthesia with thiopental, a muscle strip from the ventral costal diaphragm was dissected from the muscle *in situ.* This diaphragm strip was immediately vertically suspended in a 200 ml jacketed reservoir with Krebs-Henseleit bicarbonate buffer solution (NaCl 118 mM, KCl 4.5 mM, MgSO_4_ 1.2mM, KH_2_PO_4_ 1.1mM, NaHCO_3_ 25mM, CaCl_2_ 2.5mM and glucose 4.5 mM) prepared daily with highly purified water. The bathing solution was bubbled with 95% oxygen and 5% carbon dioxide, resulting in a pH 7.40 and maintained at 29°C.

Preparations were field-stimulated (30 V) by using two platinum electrodes with rectangular-wave pulses of 1 ms duration at 10 pulses/min in the twitch mode. After a 30 min stabilization period, at the apex of the length-active isometric force curve (L_max_), diaphragm muscle strips recovered their optimal mechanical performance [18], [19]. Measurements of mechanical variables were made at L_max_ under tetanic stimulation at 50 Hz (10 trains each minute of 300 ms duration, 1 ms rectangular pulses) to obtain maximal contractions. Cross-sectional area was calculated from the ratio of muscle weight to muscle length, assuming a muscle density of 1.06.

**Figure 1:**
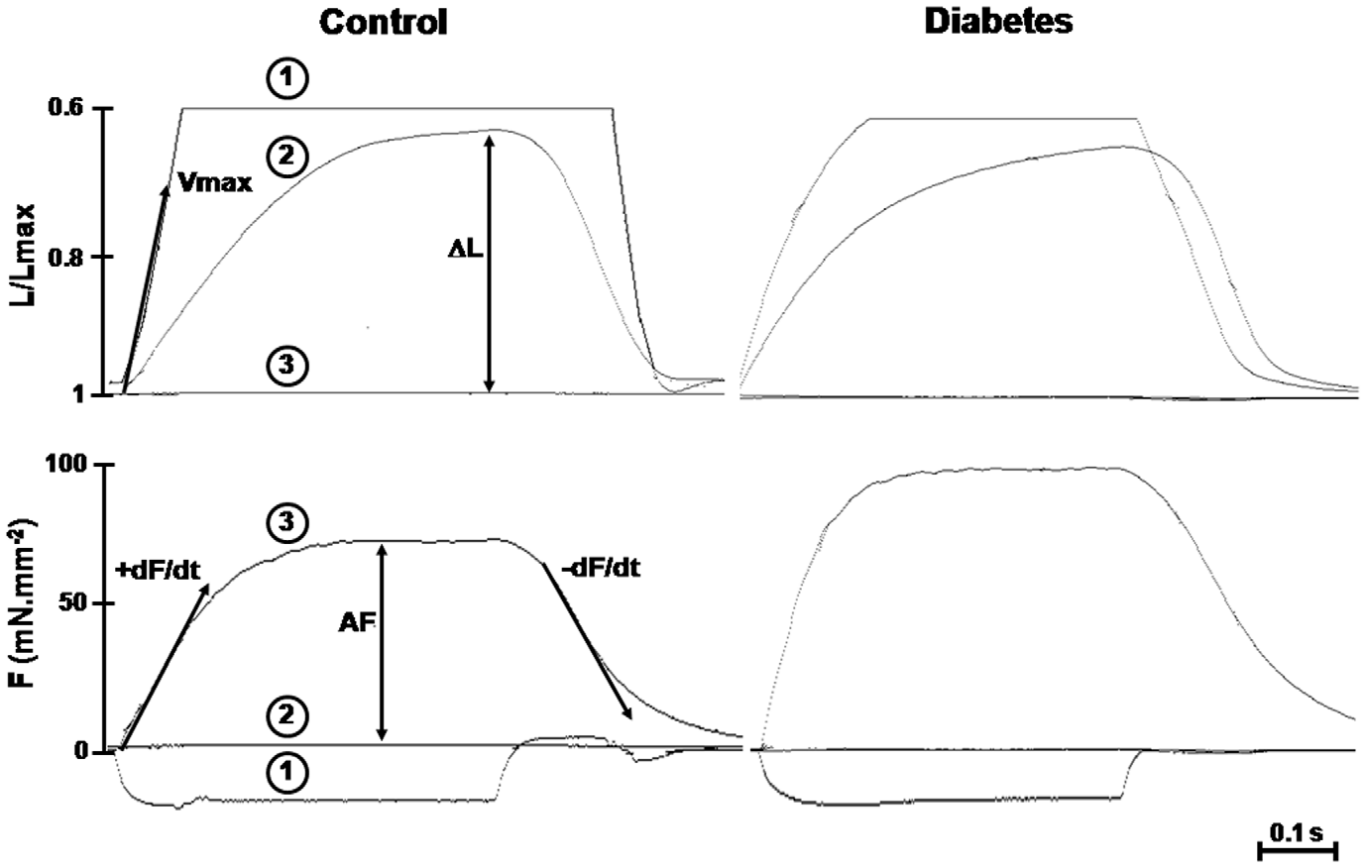
Typical traces of shortening (L/L_max_) and force (F) obtained in a control and a diabetic rat. Three consecutive tetanic (50 Hz) contractions preloaded at L_max_ with increasing afterload from zero load to fully isometric contraction are shown: 1) the first contraction was abruptly clamped to zero load, enabling determination of maximum shortening velocity (V_max_); 2) the second contraction was isotonic and loaded with preload only, enabling determination of extent of shortening (ΔL) and maximum lengthening velocity (Vr_max_); 3) the third contraction was fully isometric at L_max_, enabling determination of active force (AF)_,_ and peaks of the positive (+dF/dt^−1^) and negative (−dF/dt^−1^) derivative normalized to cross-sectional area.

The electromagnetic lever system has been described previously [20]. All the analyses were made from digital records of force and length obtained using specific software, as previously described [20], [21]. Contraction variables including the maximum shortening velocity (V_max_), the extent of shortening (ΔL), peaks of the positive derivative normalized to cross-sectional area (+dF/dt) and the active force normalized to cross-sectional area (AF) were measured. Variables characterizing relaxation including the maximum lengthening velocity (Vr_max_) and peak of the negative derivative normalized per cross-sectional area (-dF/dt^−1^) were also determined (Figure 1). Mechanical variables were calculated from 3 consecutive tetanic contractions preloaded at L_max_ with increasing afterload from zero load to fully isometric contraction. The first contraction was abruptly clamped to zero load just after the electrical stimulus, with critical damping in order to slow the first and rapid shortening overshoot resulting from the recoil of series passive elastic components, enabling determination of V_max_. The second contraction was isotonic and loaded with preload only. ΔL and Vr_max_ were determined from this contraction. The last contraction was fully isometric at L_max_. AF, +dF/dt and –dF/dt were determined from this fully isometric contraction (Figure 1). The force-velocity curve was derived from the peak shortening velocity plotted against the total force (resting force + active force) normalized per cross-sectional area [22]. Both measurements were obtained from 10 tetanic contractions, from zero load to fully isometric contraction. The total force-velocity curve was fitted according to the Hill equation [23]. The following energetic parameters were derived from the Hill hyperbola: the curvature of the hyperbola (G), non-normalized maximum power output (E_max_), and maximum mechanical efficiency (Eff_max_) [22]. The following variables of cross-bridges kinetics were determined from mechanical data with the equations of A.F. Huxley [24]: the total number of cross-bridges per mm^2^ (m), elementary force per cross-bridge (Π) and myosin ATPase activity (k_cat_).

### Effect of fatigue

After determination of baseline values, fatigue was induced by repeatedly stimulating the diaphragmatic strip with 75 trains.min^−1^ of 300 ms duration at stimulation frequency of 50 Hz. Stimulation continued until the muscle strip was fatigued to a point where it generated 45–50% of its original tetanic tension measured before the fatigue procedure. Mechanical variables were recorded just after completion of the fatigue procedure and after 20 min of recovery.

### Effect of hypoxia

Hypoxia was induced by switching the bubbling gas from 95% oxygen-5% carbon dioxide to 95% nitrogen-5% carbon dioxide leading to a fall in PO_2_ from 453±63 to 54±13 mmHg. Mechanical properties of the diaphragmatic strips were measured every 5 min over 15 min. Recovery from hypoxia was obtained by switching back to 95% oxygen-5% carbon dioxide and measurements were performed every 5 min over 15 min.

### Morphometry

Diaphragm cryosections (5 µm-width) were fixed in 4% paraformaldehyde and were incubated with mouse monoclonal anti-α actinin antibody (1/200, Sigma). Sections were revealed by anti-mouse IgG Alexa Fluor 488 (1/250, Molecular Probes, Eugene, OR) and mounted in Vectashield medium with Dapi (Vector Laboratories, Paris, France). Fluorescence was visualized using a DMRBE Leica microscope equipped with a 40x-oil epifluorescence objective. Sections exposed only to secondary antibodies were used as negative controls and showed no background staining.

### MHC isoform compositions

MHC isoform composition was determined in purified myosin as previously described [25]. In brief, sodium dodecyl sulfate-polyacrylamide (SDS-PAGE) minigel electrophoresis was performed in a mini-Protean II Dual slab cell system (Bio-Rad, Marnes-la-Coquette, France). MHC isoforms were separated on 8% polyacrylamide gels containing 30% glycerol for 24 h at 4° C and 70 V. Gels were stained with Coomassie blue. Myosin isoforms were quantified using Image Gauge software (Fujifilm, Tokyo, Japan).

### Extraction of proteins and quantification of PPARα and PPARβ/δ by Western blots

A nuclear-enriched protein fraction was extracted from frozen diaphragm fragments using a commercial kit (NE-PER nuclear and cytoplasmic extraction reagents, Pierce). Protein concentrations were determined using NanoDrop® (ND-1000 spectrophotometer, NanoDrop Products, Wilmington, DE). Samples were stored at −80°C until use. Protein samples were subjected to SDS-polyacrylamide gel electrophoresis (PAGE), transferred to nitrocellulose membranes and blotted with polyclonal anti-PPARα (1/250, SC 1985, Santa Cruz Biotechnologies, Santa Cruz, CA) and anti- PPARβ/δ (1/250, SC 1987, Santa Cruz Biotechnologies). The secondary antibody was anti-goat horseradish peroxidase (1/5000). Membranes were visualized with enhanced chemiluminescent kit (ECL, Amersham, Pantin, France). Light emission was detected with a camera (Laser 3000, Fujifilm) and quantified using Image Gauge software (Fujifilm). The S-Ponceau staining was used to verify that equal amounts of protein were loaded. Quantification of protein was normalized to actin.

### Absolute quantitative real-time PCR analysis

Total RNA was isolated from diaphragm samples using Qiagen RNeasy Kits (Qiagen France, Courtaboeuf, France) according to the manufacter's instructions. Further purification of RNA samples to remove genomic DNA was carried out with DNAse I (Roche Diagnostics, Meylan, France). The quantity of mRNA isolated from each sample was determined using the adsorption of each solution at 260 and 280 nm (ND-1000 spectrophotometer). The purity of each sample was estimated using the A260/A280 ratio and the quality checked using denatured agarose gel electrophoresis. Isolated total RNA was stored in aqueous solution at −80°C. Reverse transcription was performed on 500 ng total RNA using the SuperSript III Reverse Transcriptase (Invitrogen, Mantes La Jolie, France). Standards were generated by PCR using cDNA sub-cloned into pGEM-T-easy (Kit A1380, Promega France, Charbonières-Les-Bains, France), and all clones were verified by DNA sequencing. Specific primers for cDNAs were chosen according to the sequences available in Genbank. The sequences for PPARα were: forward, AACATCGAGTGTCGAATATGTGG and reverse, AGCCGAATAGTTCGCCGAAAG. The sequences for PPARβ/δ were: forward TTGAGCCCAAGTTCGAGTTTG and reverse, CGGTCTCCACACAGAATGATG. Real-time PCR was performed using a LightCycler (Roche Diagnostics). All real time PCR quantification were performed simultaneously with linearized plasmid standards (from 10^−3^ to 10^−6^ ng/µl) and a negative water control. The concentrations of PPARα and PPARβ/δ in the tissues were interpolated from standard curves, actin was used as a reference gene for comparative analysis.

### Statistical analysis

Data are expressed as means ± SEM. Comparisons between groups were performed using the Student's t test. All *P* values were two-tailed and a value of *P*<0.05 was considered significant. Statistical analysis was performed using NCSS 6.0 software (Statistical Solutions Ltd., Cork, Ireland).

## Results

Body weight at 8 weeks was significantly lower in diabetic compared to control rats (274±9 g vs. 337±6 g, P<0.05). Blood glucose concentrations was significantly higher in diabetic rats compared to control rats (41.1±1.2 vs.10.4±0.2 mM; P<0.05). There was no significant difference in L_max_ values between groups (10.4±0.4 vs. 9.6±0.2 mm, NS).

### Mechanical and energetic properties

**Figure 2:**
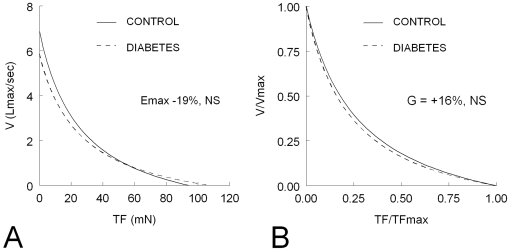
Total force (TF)-velocity (V) curves in control (n = 12) and diabetic (n = 12) diaphragms. Panel A: absolute values were used to determine the peak power output (E_max_). Panel B: the normalized TF and V values were used to determine the normalized peak power output and the curvature (G) of the F-V curve. Diabetic diaphragms were characterized by a significant increase in maximum TF (TF_max_) and a significant decrease in maximum shortening velocity (V_max_) which resulted in non significant (NS) changes in E_max_ and G.

Baseline values of mechanical and energetic parameters and cross-bridges kinetics are shown in Table 1. In diabetic rats, we observed a significant decrease in ΔL (−29%, P<0.05), V_max_ (−14%, P<0.05) and Vr_max_ (−45%, P<0.05) and a significant increase in AF (+20%, P<0.05) and +dF/dt (+41%, P<0.05). The opposite effects on force and velocity were responsible for no significant difference in the peak power output (−19%, NS) and the curvature of the force-velocity curve (-14%, NS) (Figure 2). The number of cross-bridges (m) was significantly increased (+14%, P<0.05) in diabetic rats but the force per cross-bridge remained unchanged (Table 1).

**Table 1:** Baseline main mechanical, energetic and cross-bridges kinetic variables.

	Control (n = 12)	Diabetes (n = 12)
**MECHANICS**		
ΔL (%L_max_)	41±1	29±1*
V_max_ (L_max_.s^−1^)	6.9±0.2	5.9±0.2*
AF (mN.mm^−2^)	81±2	97±1*
+dF/dt (mN.mm^−2^.s^−1^)	1862±199	2631±192*
Vr_max_ (L_max_.s^−1^)	−9.5±0.4	−5.2±0.4*
−dF/dt (mN.mm^−2^.s^−1^)	−1784±183	−1912±119
**ENERGETICS**		
G	3.5±0.2	4.1±0.4
E_max_(mN.mm^−2^.s^−1^)	44±3	36±2
Eff_max_ (%)	40±2	44±3
**CROSS-BRIDGE KINETICS**		
m (10^9^.mm^−2^)	10.1±0.4	11.5±0.3*
π (pN)	8.1±0.1	8.5±0.2
k_cat_ (s^−1^)	10.9±1.3	6.6±1.0*

**Δ**L =  maximum extent of shortening; V_max_ =  maximal unloaded shortening velocity; AF =  isometric active force normalized per cross sectional area (CSA); +dF/dt =  peak of positive force derivative normalized per CSA; Vr_max_ =  maximum lengthening velocity; −dF/dt =  peak of negative force derivative normalized per CSA; G =  curvature of the hyperbola; E_max_ =  non-normalized maximum power output; Eff_max_ =  maximum mechanical efficiency; m =  total number of cross-bridges per mm^2^; π =  elementary force developed per cross-bridge; k_cat_ =  myosin ATPase activity. Values are expressed as mean ± SEM. * *P*<0.05 *versus* control.

### Fatigue

**Figure 3:**
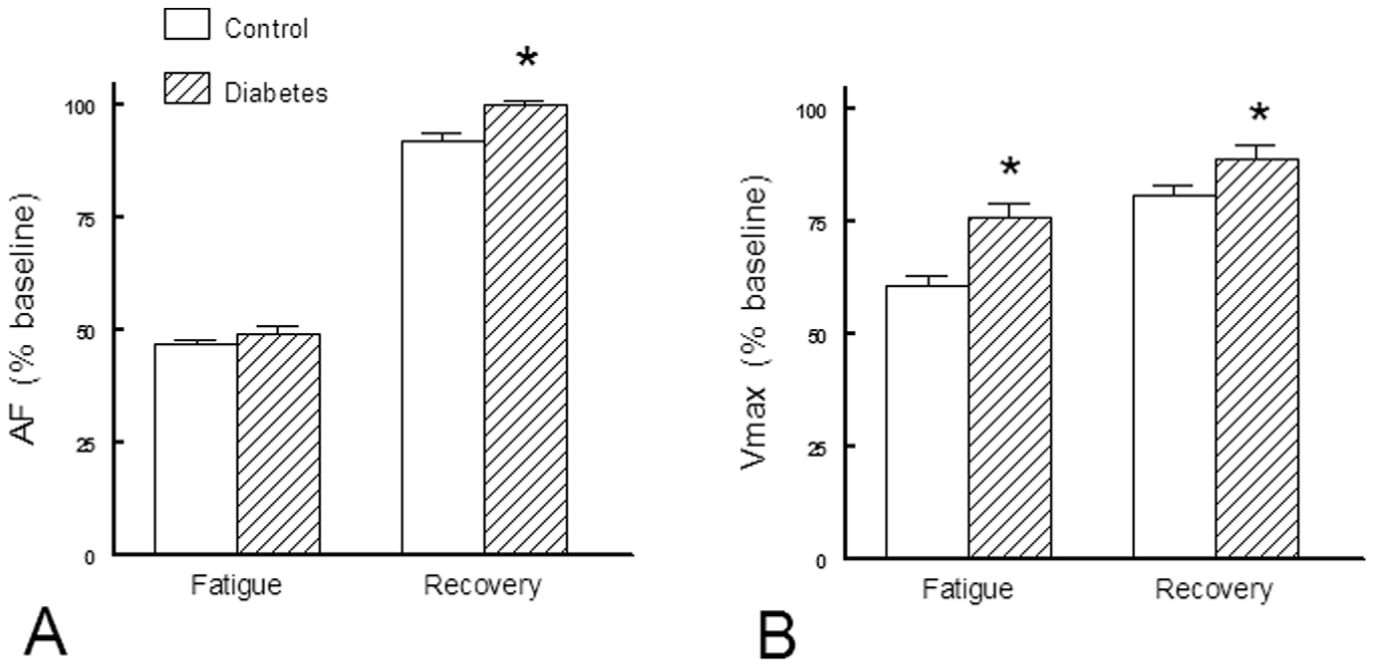
Evolution of main mechanical variables during fatigue and early recovery phase of fatigue in control (n = 12) and diabetic (n = 12) rats. Panel A: AF =  isometric active force normalized per cross sectional area (CSA). Panel B: V_max_ =  maximum shortening velocity. Values are expressed as mean percent of baseline ± SEM. *: *P*<0.05 *versus* Control.

Following the fatigue protocol, an equivalent value of AF was reached in each group but the decrease in V_max_ was less pronounced in diabetic diaphragms. Conversely, the recovery from fatigue was better in diabetic than in controls (Figure 3).

### Hypoxia

**Figure 4:**
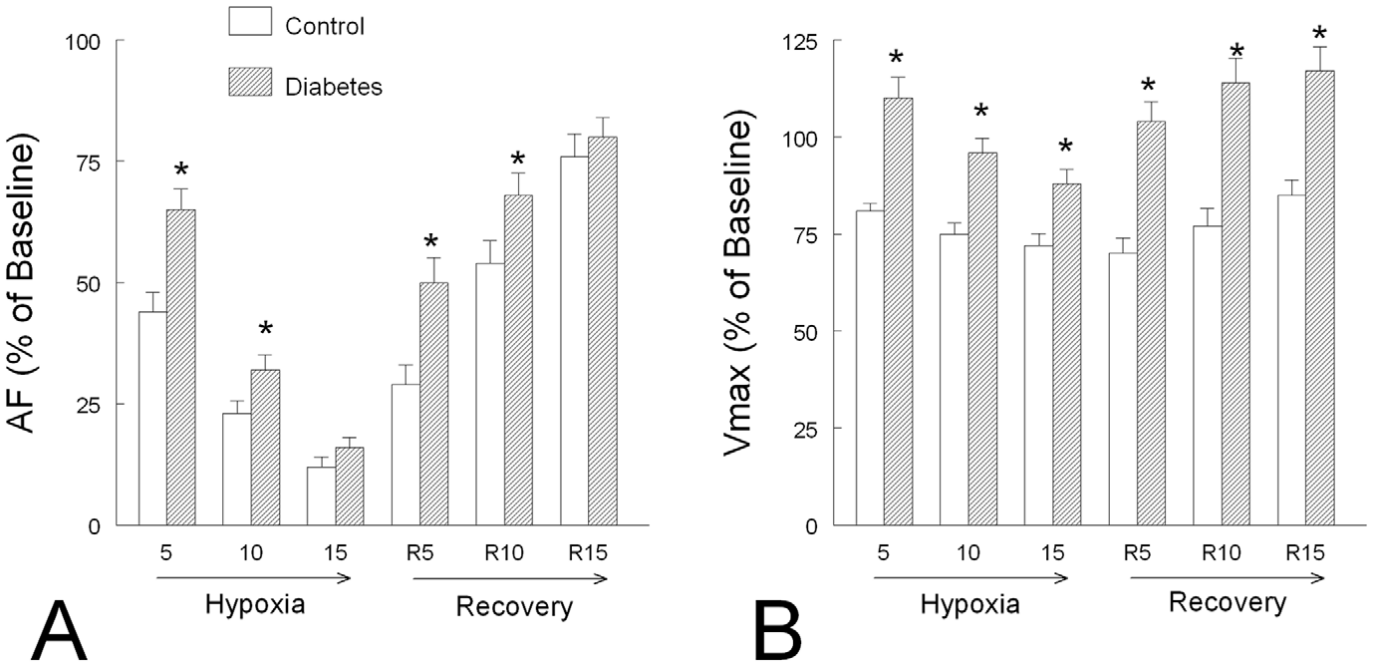
Evolution of main mechanical variables during hypoxia and recovery from hypoxia in control (n = 12) and diabetic (n = 12) rats. Panel A: AF =  isometric active force normalized per cross sectional area (CSA). Panel B: V_max_ =  maximum shortening velocity. Values are expressed as mean percent of baseline ± SEM. *: *P*<0.05 *versus* Control.

In controls, hypoxia induced a severe decrease in AF (−88%) and a less severe decrease in V_max_ (−28%). Diaphragms from diabetic rats were more resistant to hypoxia and a more rapid recovery from hypoxia was observed compared to controls (Figure 4).

### Morphometry and myosin isoform composition

**Figure 5:**
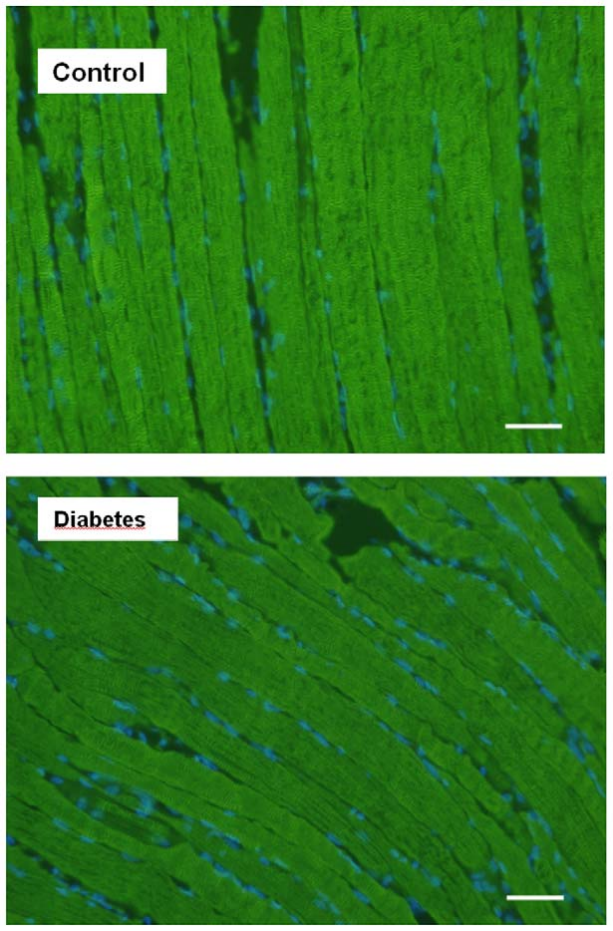
Morphometric study of diaphragm in control and diabetic rats (bar = 10 µm). Longitudinal sections are stained with α-actin antibody. Nuclei stained with Dapi appear in blue. No significant change was observed between groups.

Morphometric analysis, performed with α-actinin antibody, showed regular striation patterns in both control and diabetic rat diaphragm and did not reveal any structural abnormalities (Figure 5).

**Figure 6:**
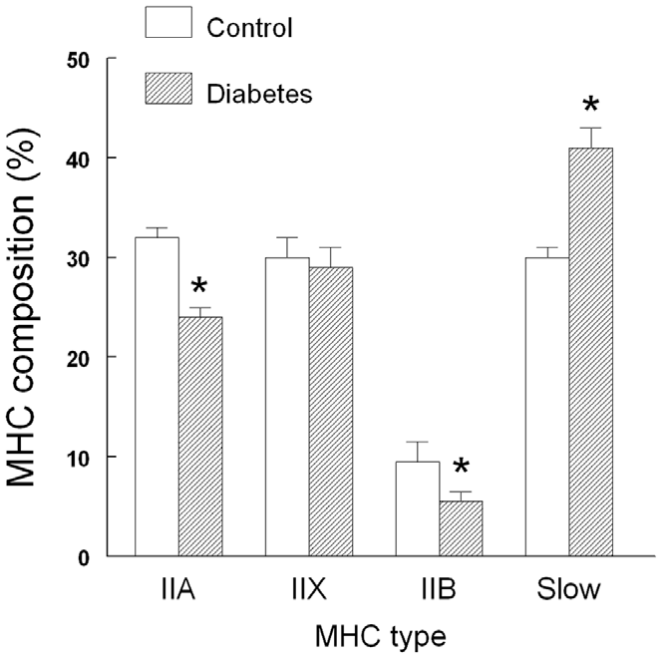
Diaphragmatic myosin heavy chain (MHC) composition in control (n = 6) and diabetic (n = 6) rats. Data are expressed as mean ± SEM. *: *P*<0.05 *versus* Control.

Four MHC isoforms were detected in the control rat diaphragm, the proportion of slow MHC-I type and overall fast MHCs isoforms being respectively ≈30% and ≈70% (Figure 6). Compared with controls, diabetic diaphragm contained a greater proportion of slow MHC fibers associated with a significant decrease in IIA and IIB MHC fibers and an unchanged proportion of IIX MHC fibers (Figure 6).

### PPAR gene and protein expression

**Figure 7:**
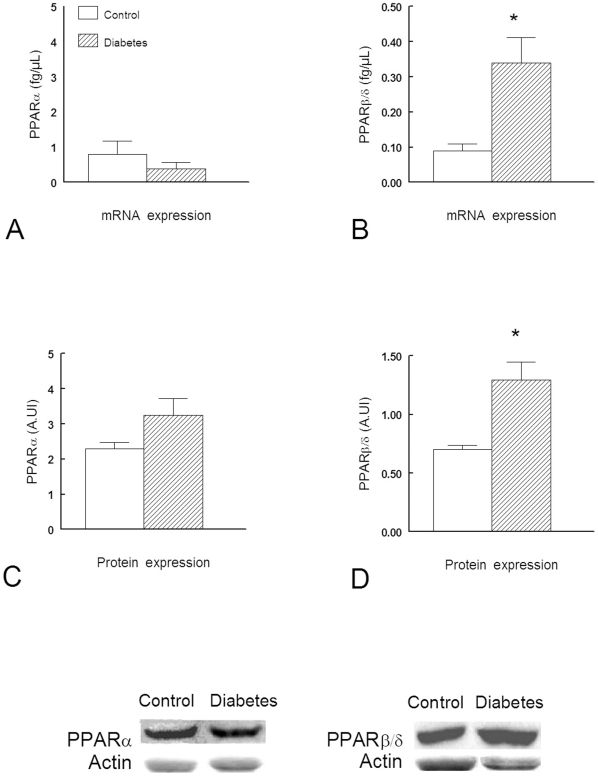
PPARα and β/δ mRNA and protein expression in diaphragm from Control (n = 6) and diabetic (n = 6) rats. *: *P*<0.05 *versus* Control. Data are expressed as mean ± SEM.

Compared to control, PPARβ/δ transcripts were significantly increased (+314%, P<0.05) in diabetic diaphragm. In contrast, the PPARα mRNA level showed no significant change compared with healthy diaphragms (Figure 7). Consistent with mRNA expression, we found that diabetes was associated with an increase in the protein expression of PPARβ/δ compared to controls (+190%, P<0.05). The protein expression of PPARα in diaphragm from diabetic rats did not significantly differ to that reported in controls (Figure 7).

## Discussion

We observed that type I diabetes induces marked changes in the rat diaphragm, mainly as a decrease in velocity of shortening associated with an increase in force, without significant change in peak power output. The analysis of cross-bridge kinetics showed a significant increase in the number of cross bridges associated with a decreased in myosin ATPase activity. These modifications were in agreement with a shift towards slow MHC fibers which were associated with an increase in PPARβ/δ. Together these modifications provided a greater resistance to fatigue and hypoxia with faster recovery in the diaphragm of diabetic rats.

There are some controversies concerning respiratory function in diabetes mellitus. Reduced vital capacity, peak trans-diaphragmatic pressures, maximum voluntary ventilation, and respiratory muscle endurance have been reported in diabetic patients [1], [2]. However, the mechanism of these changes remains a matter of debate because of the adverse effects of diabetes on peripheral airway, nerve conduction, neuroventilatory drive and perception of dyspnea [3], [4], [26], which precludes a precise analysis of respiratory muscle function *in vivo*. Streptozotocin-induced diabetes in the rat has been widely used as an animal model of diabetes mellitus [5]–[10], [16], [17] but few studies investigated the intrinsic diaphragmatic mechanical properties. McGuire et al. [11] studied the rat diaphragm after 2 months of diabetes. Although twitch tension and action potential were not modified, they reported a slight (−13%) but significant decrease in tetanic tension associated with a greater degree of fatigue, and highlighted noticeable differences between diaphragm and other skeletal muscles [27]. Compared with our study, the only experimental differences were the use of isometric twitch contraction during the whole procedure. Hida et al. [8] also observed a significant decrease in diaphragmatic contractility in diabetic rats which could be reversed by treatment with N-acetylcysteine but this study is difficult to interpret since only a very short period of diabetes (3 and 7 days) was induced. In contrast to these studies, we observed that, although velocity of shortening was slightly decreased, force was increased, without significant change in peak power output. Moreover, our results concerning mechanics, energetics, fatigue, MHC composition, and PPAR isotypes, are consistent overall.

The rat diaphragm is a composite striated muscle which contains approximately 40% of slow type I fibers, 27% of type IIA, and 33% of rapid type IIB fibers [28]. The shift toward slow MHC fibers reported here (Figure 6) is consistent with the observed decrease in shortening velocity and myosin ATPase activity (Table 1). It is widely accepted that the expression of MHC isoforms correlates with shortening velocity and myosin ATPase activity, the MHC_slow_ isoform displaying the lowest shortening velocity and myosin ATPase activity [29]. However, fibers expressing MHC_slow_ generated less maximum specific forces than fibers expressing MHC_IIX_ or MHC_IIA_ although their calcium sensitivity is considered to be higher [29]. Therefore, this fast-to-slow fiber shift reported in our study was also associated with an increase in force and the number of cross-bridges (Table 1), explaining why the peak power output and the curvature of the force-velocity curve remained unchanged (Figure 2). The lack of change in force per cross-bridge we observed (Table 1) is due to the fact that the unitary force per cross-bridge is similar across MHC isoforms and that differences in specific forces reflect differences in MHC content per sarcomere [29]. The relative increased proportion of fibers expressing the MHC_slow_ isoform may explain the greater fatigue resistance of diabetic diaphragm [30]. It has been shown that fatigue-resistant fibers comprise those expressing the MHC_slow_ and MHC_IIA_ isoforms and that they are characterized both by a low myosin ATPase activities and a high succinate dehydrogenase activity which reflects the overall aerobic capacity of muscle fibers [31]. The fast-to-slow fiber shift may also explain the greater hypoxia resistance of diabetic diaphragm since it has also been shown that slow fibers are also less sensitive to hypoxia [32]. Nevertheless, we cannot ruled out the hypothesis that other associated mechanisms were involved particularly because modifications inducing resistance to hypoxia may occur without significant changes in myosin heavy chain isoform [33].

PPARs belong to a family of nuclear receptors that regulate fatty acid metabolism via ligand-dependent transcriptional activation of target genes. PPARα mediates lipid-induced activation of fatty acid β-oxidation and plays a central role in the metabolic and energetic homeostasis of striated muscles in which it is abundantly expressed, including the diaphragm [34]. In PPARα knockout mice, diaphragmatic muscle mechanics, energetics, and cross-bridge kinetics are markedly impaired, leading to histological injuries [34]. PPARβ/δ expression predominates in heart and skeletal muscle, including the diaphragm [15]. PPARβ/δ has been shown to engineer the fast-to-slow fiber shift induced by endurance training [35]. In our study, we observed an increase in gene and protein expression of PPARβ/δ without significant changes for PPARα. These results are consistent with those of Van Lunteren et al. [10] who found a global increase in expression of genes involved in lipid metabolism in the diaphragm of type I diabetic rats. Since PPARβ/δ drives the synthesis of type I muscle fibers [12], it is likely that it played a crucial role in the mechanical and energetic changes induced by diabetes in the rat diaphragm. PPARα and PPARβ/δ direct distinct metabolic regulatory programs and PPARα mice develop lipotoxic cardiomyopathy which resembles the diabetic cardiomyopathy whereas PPARβ/δ mice do not [36]. In contrast to that we observed in the diabetic diaphragm, Yu et al. [17] observed a decrease in PPARβ/δ gene and protein expression in the myocardium of diabetic rats whereas Huang et al. [37] observed an increase in PPARβ/δ gene expression in the lung. Taken together, these results suggest that PPARβ/δ expression in diabetes differs markedly from one tissue to another [37], [38], possibly depending on the balance between the two major fuel utilization pathways, glucose versus fatty acid and their cross-talk. Since PPARβ/δ has been suggested as a potential target for metabolic modulation therapy aimed at diabetes-induced cardiomyopathy [36], our study may signal possible adverse effects related to this differential expression of PPARβ/δ in different tissues during diabetes. Interestingly, we did not find significant changes for PPARα, whereas increased PPARα expression has been reported in diabetes type II animal models [39]. Such differences support the hypothesis that early stages of obesity and insulin resistance are accompanied by increased rather than reduced β-oxidation [39].

The following points should be considered when assessing the clinical relevance of our results. First, this *in vitro* study only dealt with intrinsic diaphragmatic contractility. Observed changes in diaphragmatic function *in vivo* in diabetes depend also on modifications in diaphragmatic arterial blood flow, central nervous system respiratory drive and neuromuscular transmission. Second, this study was lead at 29°C and at low-frequency stimulation. However, diaphragmatic muscle must be studied at low temperature because stability of mechanical parameters is not sufficient at 37°C, and they must be studied at low-frequency because high-frequency stimulation induces core hypoxia [40]. The experimental model used is thought to mostly reflect type I diabetes mellitus whereas type II diabetes mellitus is usually studied using genetic models such as Zucker rats [41]. It should also be pointed out that our study was conducted in animals with uncontrolled diabetes whereas most diabetic patients receive some form of therapy. The diabetes-induced changes were significant after 8 weeks of diabetes. This result is consistent with our previous observation in the diabetic rat myocardium [16], [17], and emphasizes the importance of the duration of exposure to diabetes in experimental models and thus to differentiate models mimicking acute hyperglycemia [8] and those mimicking diabetes [41]. Although the diaphragmatic function was preserved in our model, the long-term consequences of at least some of these adjustments may finally impaired muscle function, for example, if there is increased mitochondrial biogenesis with concomitant oxidative stress. Lastly, this study was performed in the rat and species differences cannot be excluded although MHC quota is quiet similar in rat and human diaphragms [28], [42].

In conclusion, in rat diaphragm, diabetes type I induced complex mechanical and energetic changes, associated with improved resistances to fatigue and hypoxic condition that may be explained mainly by a shift towards slow MHC isoforms associated with an increased expression of PPARβ/δ gene and protein.
